# Kinetic interpretation of log-logistic dose-time response curves

**DOI:** 10.1038/s41598-017-02474-w

**Published:** 2017-05-22

**Authors:** Walter W. Focke, Isbe van der Westhuizen, Ndeke Musee, Mattheüs Theodor Loots

**Affiliations:** 1 Institute of Applied Materials, Department of Chemical Engineering, University of Pretoria, Pretoria, South Africa; 2 Department of Statistics, University of Pretoria, Pretoria, South Africa

## Abstract

A Hill-type time-response curve was derived using a single-step chemical kinetics approximation. The rate expression for the transformation is a differential equation that provides an interpolation formula between the logistic growth curve and second order kinetics. The solution is equivalent to the log-logistic cumulative distribution function with the time constant expressed in terms of a kinetic rate constant. This expression was extended to a full dose-time-response equation by postulating a concentration dependence for the rate constant. This was achieved by invoking a modified form of Haber’s law that connects an observed toxic effect with the concentration of the active agent and the elapsed exposure time. Analysis showed that the concept of Concentration Addition corresponds to a special case where the rate constant for the overall transformation rate is proportional to the sum of the rate constants that apply when the agents act individually. Biodiesel “survival” curves were measured and used to test the applicability of the empirical model to describe the effects of inhibitor dosage and binary inhibitor mixtures. Positive results suggest that the proposed dose-response relationship for the toxicity of agents to organisms can be extended to inanimate systems especially in cases where accurate mechanistic models are lacking.

## Introduction

The performance of real-world systems are governed by numerous interacting subsystems. Exact mechanistic modelling is the preferred approach but this requires comprehensive knowledge of the interrelationships and interactions between all the elements of the functioning system. The complexity involved is often almost beyond measure and, especially for biological phenomena, cannot be predicted at the level of numerical precision achievable in classical physics^1^. Such complexity extends to inanimate systems. The mechanisms of processes in condensed phases tend to occur in multiple steps at different rates. They are often unknown or too complex to be characterised by a simple model that provides exact predictions. For example, even a simplified mechanistic kinetic model for the thermal oxidation of an unstabilised, saturated hydrocarbon polymer, driven by hydroperoxide decomposition, requires experimental evaluation of a large number of temperature-dependent rate constants^2^. Fortunately, according to von Bertalanffy’s General Systems Theory^3^ universal principles apply to such complex systems. The overall dynamic behaviour can often be approximated by a much simpler model. For instance, single-step reaction approximations were found useful to describe condensed phase kinetics, albeit at the expense of insights into the underlying mechanisms^4^. Nevertheless, the single-step kinetics approach yields a useful tractable mathematical description as a workable substitute for the generally complex set of kinetic equations^4^.

Dose-response and time-response equations are widely employed in diverse scientific fields^5–8^. In the most general case, they are used as combined dose-time-response models. They are employed to describe the temporal variation of a representative magnitude in an object population, as influenced by variation in the magnitude of an effector agent^5–9^. Depending on the field, the “population” may represent humans, other biological lifeforms in ecological systems, farm lands, chemical compounds, enzymes, etc. Dose-response models are used to describe the effect of the amount of a toxin or a therapeutic drug on survival in biological communities; the combined effect of inhibitors on enzymes^10^; the relationships among exposure time, concentration, and toxicity of insecticides^11^; the quantity of applied fertiliser on agricultural yield^12^; the relationship between herbicide dose and plant response^13^; the decomposition kinetics^14^ or the polymorphic transformations of solids^15^, and the concentration of antioxidants on the oxidative stability of organic materials, etc.

**Figure 1:**
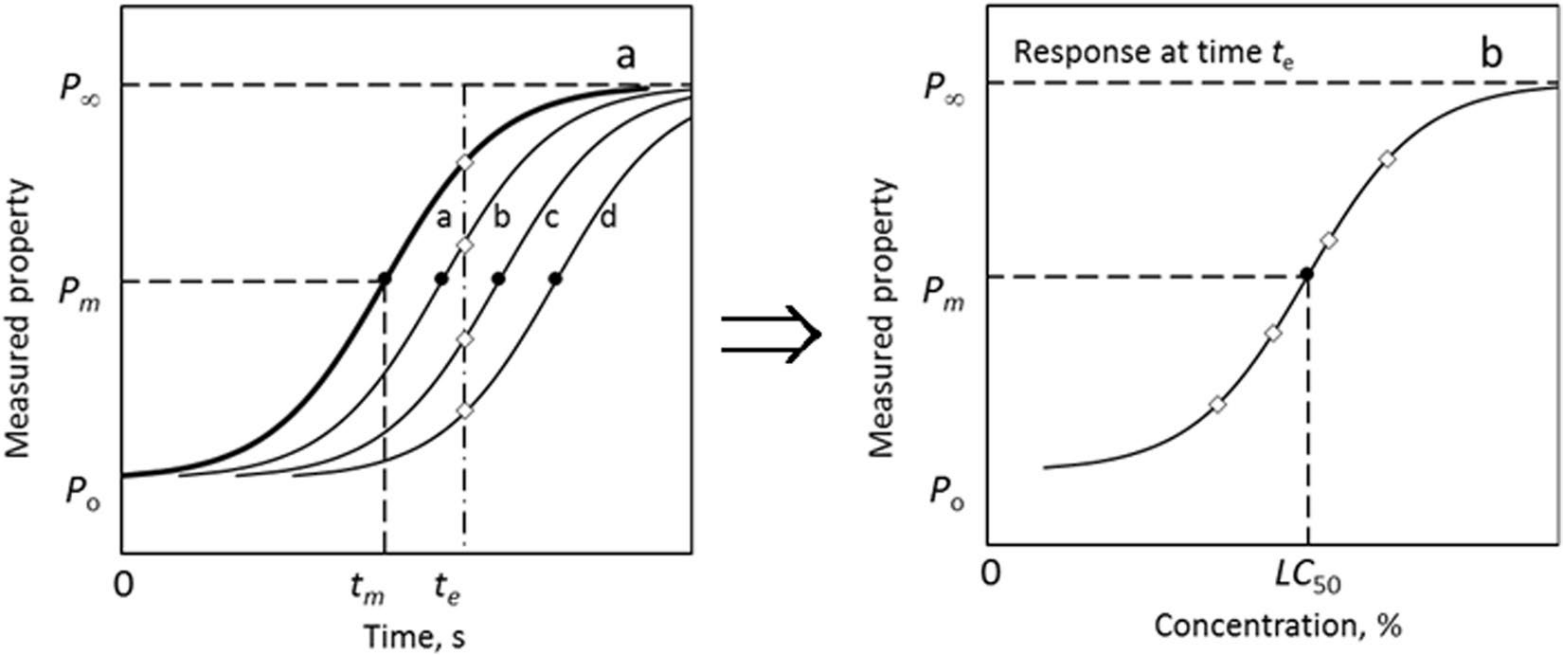
(**a**) Schematic representation of time-response curves, showing the temporal evolution of a measurable property *P* (e.g. mortality in biological systems) following the logistic curve for different active agent dose levels. The concentration or dose level either increases or decreases in the order: a < b < c < d. The magnitude of the property ranges from *P*_o_ at zero transformation to *P*_∞_ at complete transformation (or conversion). The property of central interest is the time *t*_m_ where the transformation equals the median effect value *P*_*m*_ = (*P*_∞_ − *P*_*o*_)/2. (**b**) Dose response curve for a fixed elapsed treatment time derived from the set of time-response curves. The median response is attained at a dosage equivalent to *LC*_50_.

Notably, the response functions usually exhibit a sigmoidal time- or dose dependence as indicated in Fig. 1. The logistic equation, also known as the Boltzmann sigmoidal model, is the classic example of a sigmoidal function. It has a long history and has been used to model diverse scenarios including animal and human population ecology^16–18^, bioassays, radioreceptor assays and radioimmunoassays^19, 20^, and also chemical reactions^21, 22^. The logistic equation^21^ assumes that the transformation rate is proportional to both the transformed and the untransformed substrate. It predicts that the transformation initially proceeds exponentially, then slows and eventually saturates to produce the characteristic S-curves shown in Fig. 1. Both the time-response and the dose-response curves tend to show sigmoidal trends.

The sigmoidal response time-response and dose-response curves provide an overview of the performance of the activity of the effector agent under consideration. In most applications, e.g. in toxicity studies, the number of experiments are limited by time and cost constraints and, in particular, by ethical considerations. It is therefore difficult to generate the numerous data points necessary for full elucidation of the expected sigmoidal curves. However, often complete knowledge is not essential. This is particularly true in toxicity studies. It may be sufficient to accurately estimate the dose or concentration of the active agent that will result in the median response. For example, *LD*_50_ is the lethal dose that will kill 50% of the population of a specific test organism in a given period of time; while the *LC*_50_ is the lethal concentration required to kill 50% of the population studied.

Nevertheless, assuming the validity of the universality of complex logistic-like behaviour, it is of interest to study proxy systems that do allow generation and analysis of more comprehensive data sets. For the purposes of this study, biodiesel stabilization was investigated as a proxy system. The biodiesel was taken to represent a substitute substrate (or population) that is subjected to a chronic stress factor, which in this case, entailed exposure to air at an elevated temperature. The progress of the demise of the substrate was conveniently and continuously measured using a Thermomat. The measured response followed a sigmoidal curve that indicated the intrinsic stability of the substrate in the form of a proxy life-time curve. The curve was found to shift towards longer times when suitable antioxidants were added. Figure 1 shows this schematically. Figure 1b shows that, for a fixed evaluation time, the response curve describing the effect of the antioxidant concentration on performance is also sigmoidal. We posit that this (or such a) proxy system is analogous to the expected behaviour of an arbitrary population subjected to a chronic stress factor, e.g. a toxic chemical in the environment. The antioxidant simulates the effect of a once-off dose of a therapeutic agent that assists the individuals in the population to cope with the chronic stress and therefore survive for a longer time. The net result is an extension of the median “life expectancy”.

Therefore, this communication considers the general case of a substrate/population that is subjected to a chronic stress factor for which an effector agent can provide partial relief. The objective is to develop the simplest mathematical model capable of simultaneously describing the effects of both time and the concentration of the effector agent. This model is then applied to survival curves measured for antioxidant-stabilised biodiesel as a proxy for real life systems. The effect of binary antioxidant mixtures is also considered. The focus of this communication is on estimating the median response from such experimental sigmoidal curves and to determine how it varies with time and concentration of the antioxidants.

## Results

### Dose-time survival curve model

The Hill survival function was obtained by solving a parametric differential equation that describes the rate of transformation (or conversion in chemical terms) of the substrate:

1 $$S(t)=1/[1+{(t/\tau )}^{\theta }]$$

Here *S*(*t*) is the survival (survivor or reliability) function which is the probability that the “substrate of interest” will survive beyond a specified time *t*; *τ* is the time constant and *θ* is a shape factor. A convenient property of the Hill equation is the fact that the constant *τ* corresponds to the time where the transformation reaches the half way mark, i.e. the median response. The rate constant of the differential equation, and therefore also the time constant *τ*, must be dependent on the concentration of the active agent. The necessary link was made via the modified Haber rule^5, 11, 23, 24^ yielding

2 $$\,\tau ={[\kappa {(C-{C}_{o})}^{\beta }]}^{-1}$$

The Plackett and Hewlett^25, 26^ generalization for the action of two agents administered simultaneously lead to the following expression:

3 $${\tau }_{mix}={[{{\tau }_{A}}^{-1/\beta \lambda }+{{\tau }_{B}}^{-1/\beta \lambda }]}^{-\beta \lambda }$$

where *λ* is a constant, *τ*_mix_ is the effective time constant for the mixture and the *τ*_*i*_ values apply for the cases when the agents were used on their own.

### Application to biodiesel oxidation

**Figure 2:**
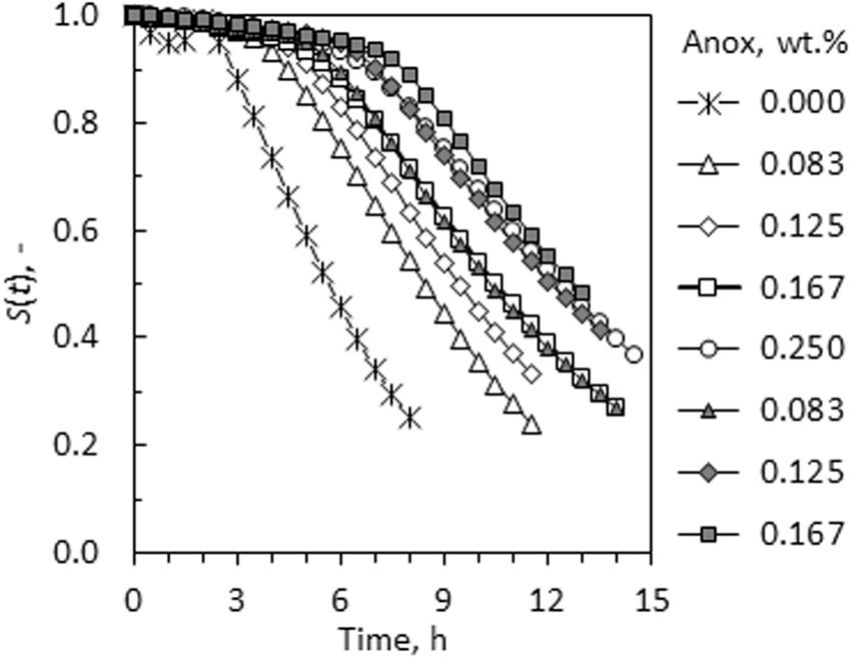
Experimental biodiesel “survival curves” derived from conductivity-based time-response data. Open symbols: Neat Anox 20 at different dosage levels. Filled symbols: Mixtures of Anox 20 and Orox PK with the total antioxidant dose held at constant at 0.25 wt.%.

Figure 2 shows selected experimental survival results for neat sunflower biodiesel as well as samples spiked with antioxidants. The curves are typical examples that show the effect of concentration on the time-response for Anox 20 (single antioxidant), and a binary mixture of the antioxidants Anox 20 and Orox PK. For the binary antioxidant mixtures, the total amount of antioxidant was kept constant at 0.25 wt.%. Least square regression over all the experimental data sets determined *θ* = 3.45 ± 0.10.

**Figure 3:**
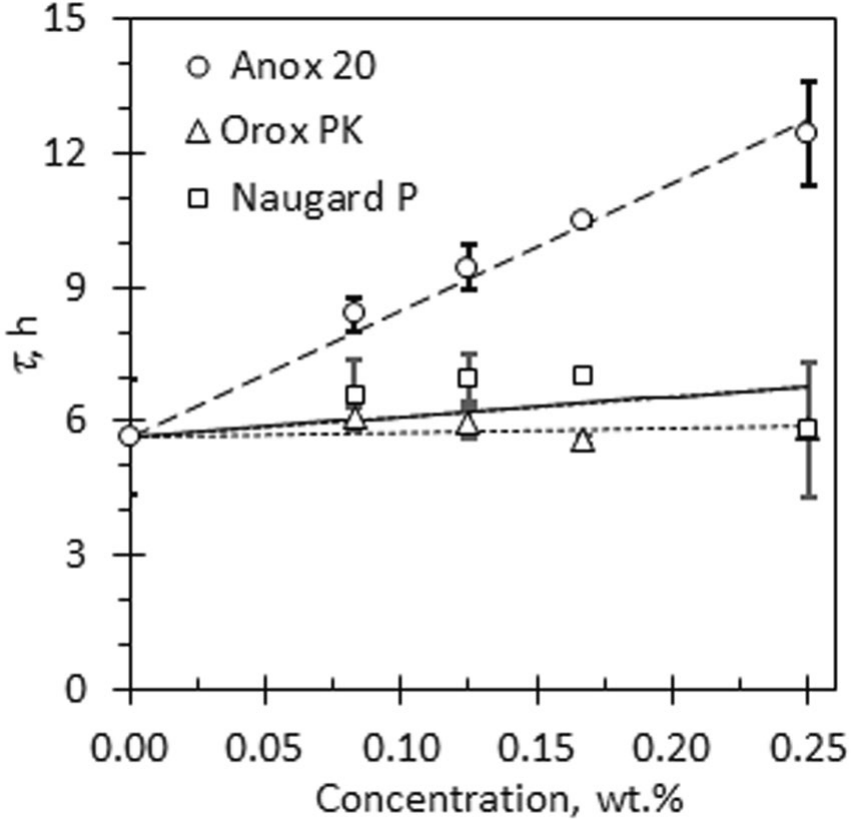
Variation of time constant *τ* with neat antioxidant concentration. Error bars indicate ± 3 standard deviations.

**Figure 4:**
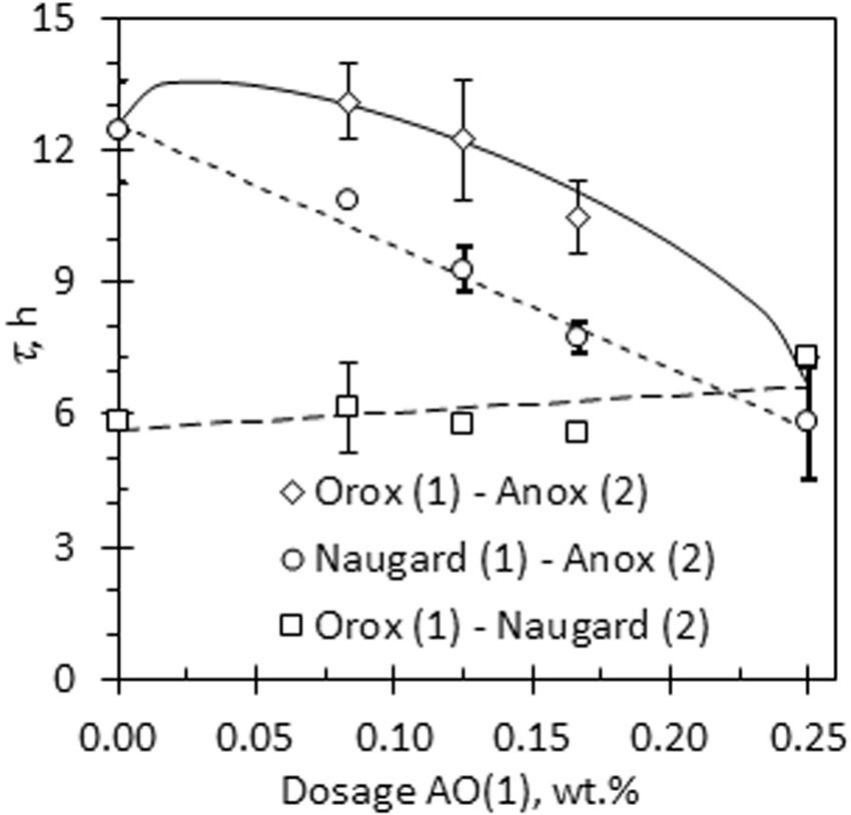
Variation of the time constant *τ* with antioxidant dosage for binary antioxidants mixtures. The total dose of antioxidant dose was held constant at 0.25 wt.%. Dosage reported in the graph is for the first antioxidant in the binary mixture.

Figure 3 shows how the time constant *τ* varies with single antioxidant concentration. Figure 4 illustrates the variation of the time constant *τ* with antioxidant binary blend composition when the total concentration is kept constant at 0.25 wt.%. The results suggest that it was possible, by adding a binary mixture of Anox 20 and Orox PK, to more than double the *τ* value; thus signifying enhanced stability of the biodiesel (Table 1).

**Table 1:** Results of neat antioxidants and binary mixtures of the antioxidants.

Sample	Orox PK	Naugard P	Anox 20	*τ*, h (exp.)	*τ*, h (fitted)	Difference, %
Biodiesel	0	0	0	5.66 ± 0.43	5.66	0.00
Single antioxidants						
Anox 20	0	0	0.083	8.39 ± 0.12	8.01	4.5
	0	0	0.125	9.45 ± 0.16	9.20	2.6
	0	0	0.167	10.99 ± 0.04	10.39	1.0
	0	0	0.250	12.42 ± 0.38	12.74	2.6
Naugard P	0	0.083	0	6.12 ± 0.25	5.74	6.2
	0	0.125	0	5.95 ± 0.19	5.77	3.0
	0	0.167	0	5.57 ± 0.01	5.81	4.3
	0	0.250	0	5.82 ± 0.50	5.88	1.0
Orox PK	0.083	0	0	6.60 ± 0.06	6.03	8.5
	0.125	0	0	6.96 ± 0.12	6.22	10.6
	0.167	0	0	7.00 ± 0.03	6.41	8.5
	0.25	0	0	5.82 ± 0.05	6.78	16.4
Binary mixtures	0	0.167	0.083	7.75 ± 0.48	8.16	5.3
	0	0.125	0.125	9.30 ± 0.93	9.31	0.1
	0	0.083	0.167	10.86 ± 0.00	10.45	3.7
	0.167	0.083	0	5.59 ± 0.00	6.48	15.8
	0.125	0.125	0	5.75 ± 0.01	6.33	10.0
	0.083	0.167	0	6.14 ± 0.34	6.18	0.7
	0.167	0	0.083	10.46 ± 0.28	11.41	9.1
	0.125	0	0.125	12.25 ± 0.46	12.56	2.5
	0.083	0	0.167	13.11 ± 0.29	13.40	2.2

Notes: 1. Compositions are in units of wt.%; and 2. “Neat” refers to biodiesel without any added antioxidant. 3. Fitted using equations (4) or (5).

## Discussion

The purpose of this study was not to develop an understanding of the operating antioxidant mechanisms and their interactions. Therefore, the comments made here are limited to straight forward observations. Of the three antioxidants tested, Anox 20 was the most effective as it increased the stability of the biodiesel. Naugard P, for all practical purposes, showed no stabilising effect. Figure 3 indicates that, in the range of antioxidant concentrations tested, the time constant varied approximately linearly with dosage:

4 $$\tau ={\tau }_{o}+{\kappa }_{i}{C}_{i}$$

where *τ*_*o*_ is the time constant for the neat biodiesel and *τ*_*i*_ = *κ*_*i*_ *C*_*i*_ is the time constant contribution by the antioxidant *i* dosed at a concentration *C*_*i*_. This implies that *β* = −1 for the current antioxidants used to stabilise the biodiesel. Equation (4) also holds for the binary blends Anox 20 + Naugard P and Orox PK + Naugard P. See Fig. 4. However, this was not the case for binary blends of Anox 20 with Orox PK as the results revealed a strong synergistic interaction between these two antioxidants (Fig. 4). The plotted line for this binary blend in Fig. 4 corresponds to a fit to the Plackett and Hewlett^25, 26^ equation (27) with *λ* = 2, i.e.

5 $${\tau }_{mix}={\tau }_{o}+{(\sum _{i}\sqrt{{\tau }_{i}})}^{2}$$

Clearly there is reasonable agreement between model and experimental data.

This study followed a kinetic approach to establish a general mathematical framework for the temporal response of a system or substrate being transformed by the action of one, or a combination of active agents administered at different dosage levels. The effect of an environmental factor on the rate of transformation of the system was also considered. Herein the system may imply a human, a biological lifeform in an ecological system, an agricultural farm, a chemical formulation, or a material among others. The active agents may include drugs, inhibitors, toxins, fertilisers, biocides, pesticides, weed killers, etc. The effect of an environmental factor, in this case temperature, on the rate of transformation of the system was also considered.

As proxy system, the stabilisation of sunflower biodiesel against oxidative degradation was used as an illustrative example. The fuel was fortified by addition of antioxidant combinations in order to extend its useful temperature-dependent service life. The stability of the biodiesel was evaluated using the Thermomat method. The experimental response curves were fitted to the Hill-equation equivalent to a log-logistic distribution. This allowed facile evaluation of the time constant *τ*, the time to the median response, used in this case as quantifier for the oxidative stability of the biodiesel. It was found to vary linearly with antioxidant concentration when they were added alone. The strong synergistic interaction between the antioxidants Anox 20 and Orox PK was adequately described by the Plackett and Hewlett^25, 26^ equation. Although this equation was originally developed for applications in toxicity studies, here it was found to be applicable to account for the “survival” of biodiesel. These results supports Bertalanffy’s idea that universal principles hold true to diverse complex systems^3^. Therefore the ideas presented in this communication can potentially find widespread application since the specific biodiesel example can be considered as a proxy for more general cases. Indeed, the development of the proposed model was informed by concepts established in widely different fields.

## Conclusions

A Hill-type time-response function follows from the assumption that the transformation of a substrate, under the influence of an effector agent, is described by an autocatalytic rate expression. A full time-dose-response relationship is obtained if the kinetic rate constant is made to be dose-dependent. This can be justified by invoking a modified form of Haber’s rule that defines the relationship between exposure time and the concentration of a toxic agent.

According to the resulting time-dose-response model, the concept of Concentration Addition corresponds to the special case where the rate constant, for the overall transformation rate, is equivalent to the sum of the rate constants that apply when the agents act individually.

The utility of the empirical model, to describe the effects of inhibitor dosage and binary inhibitor mixtures, was tested using experimental biodiesel “survival” curves. Positive results suggest that the proposed dose-response relationship for the toxicity of agents to organisms can be extended to inanimate systems especially in cases where accurate mechanistic models are lacking.

## Methods

### Theoretical method

In the context of chemical reaction kinetics, the logistic rate equation is defined as

6 $$\frac{d{\rm{\alpha }}}{dt}=k{\rm{\alpha }}(1-{\rm{\alpha }})$$

where *k* is the rate constant *α* is the degree of conversion. Equation (6) is also referred to as the Prout-Tompkins rate equation^22, 27^. In chemistry it represents the archetype autocatalytic reaction with a well-defined induction time that is described by a single controlling transformation variable *α*. This is equivalent to the “conversion” in chemical terminology. An autocatalytic reaction is one for which at least one of the reaction products acts as a catalyst to accelerate the course of the reaction. This type of behaviour has been observed in the decomposition kinetics of inorganic solids^27^, explosives^28^ and in resin cure reactions^29^. Many chemical systems appear to follow autocatalysis when several chemical species participate through a complex set of primitive reactions. A good example is the oxidation of hydrocarbons and polymers which proceeds as an autocatalytic, free radical chain reaction process^30^. The conversion curve displays an induction time and features a sigmoidal shape^2^. While a sigmoidal response curve appears to be an intrinsic property of this system, it can be shifted to longer times by addition of antioxidants^31^. Analogous examples are found in explosives technology^32^.

When an autocatalytic reaction defined by equation (6) proceeds isothermally, the general solution is:

7 $${\rm{\alpha }}={e}^{k(t-{t}_{o})}/[1+{e}^{k(t-{t}_{o})}]\,or\,1-{\rm{\alpha }}=1/[1+{e}^{k(t-{t}_{o})}]$$

Here *t*_o_ serves the purpose of the required integration constant. In theory it can be determined from knowledge of the degree of conversion at time *t* = 0:

8 $${t}_{o}=[\mathrm{log}(1{/{\rm{\alpha }}}_{o}-1)]/k$$

Note that the degree of conversion attains the value *α* = 0.5 at *t* = *t*_o_. It is usual to assume that, for chemical systems, the rate constant *k* follows an Arrhenius temperature dependence.

On occasion it is not the degree of conversion per se that is of interest but rather some measureable physical property *P* that depends on the state of transformation of the system. In chemical systems the latter corresponds to the degree of conversion of the limiting reagent. The simplest dependence would be a direct proportionality. Thus let *P*_o_ and *P*_∞_ correspond to the states *α* = 0 and *α* = 1 respectively. Then variation of the property *P* with time can be written in the following two equivalent forms:

9 $$P(t)={P}_{o}+({P}_{\infty }-{P}_{o}){\rm{\alpha }}={P}_{\infty }+({P}_{o}-{P}_{\infty })(1-{\rm{\alpha }})$$

The important physical property of concern in the present study was the change in the conductivity of water used to trap the vapours emitted by biodiesel degrading during the oxidation experiments.

More generally, the degree of conversion, i.e. the variable *α*(*t*), is a cumulative statistical distribution function *F*(*t*) defined on the real line. In practice it is often only the positive domain that is of interest. In this regard, empirical evidence has shown that the considerable flexibility of the log-logistic distribution allows it to deal with the complex interactions controlling many situations. This includes applications in toxicity^33^, in hydrology^34^, income distribution in economics (where it is known as the Fisk distribution^35^), autocatalytic cure reactions^29^ or solid state decomposition reactions based on nucleation and growth mechanisms in chemistry^14^. In pharmacology, medicinal chemistry and biology the log-logistic distribution is known as the median-effect or Hill equation^36, 37^. It is, for instance, widely used to model response surfaces in drug combination studies^38^ including multiple anaesthetic drugs^39^; the effect of fungicide combinations^40^; the inhibition of cancer cell growth in the joint action of multiple anticancer agents^41, 42^, the interactions among toxicants^43^ and the effects of multiple inhibitors on enzyme systems^38^.

The Hill equation was originally derived on the basis of equilibrium principles applied to the cooperative binding of ligands to a macromolecule^44^. Instead, for the present discussion, it is fruitful to consider the Hill equation to arise from the following rate expression that governs transformation of the substrate:

10 $$\frac{d\alpha }{dt}=k{\alpha }^{1-1/\theta }{(1-\alpha )}^{1+1/\theta }$$

where the constant *θ* is a shape parameter. Note that this differential equation provides a parametric interpolation formula between the predictions of the logistic equation (*θ* → ∞) and second order kinetics (*θ* = 1). The general solution is

11 $${[\alpha /(1-\alpha )]}^{1/\theta }=1+[k(t-{t}_{o})/\theta ]$$

It can be cast in the following explicit form:

12 $$1-\alpha =1/\{1+{[1+k(t-{t}_{o})/\theta ]}^{\theta }\}$$

For *θ* → ∞ Equation (10) reduces to equation (6). For 0 < 1/*θ* < ∞ it is possible to force *α* = 0 at *t* = 0 by setting *t*_o_ = ﻿0. With this condition the equation reduces to the simpler form:

13 $$1-\alpha =1/[1+{(t/\tau )}^{\theta }]$$

where the time constant, defined by *τ* = *θ*/*k*, is a scale parameter. The distribution function *F*(*t*) = *α*(*t*) is the general Hill time-response equation that describes the temporal transformation of the population. It is the probability that, at a fixed time *t*, the transformation of the substrate has proceeded to an extent equal to or less than *α*(*t*). The odds for this happening is the chance of it occurring and this is given by the ratio *α*/(1−*α*):

14 $${\rm{\alpha }}/(1-{\rm{\alpha }})={(t/\tau )}^{\theta }$$

The question is now how to go about transforming this empirical equation into an effective dose-time-response relationship for mixtures of active agents. The key is provided by the parameter *τ*. It is the median of the log-logistic distribution, i.e. it corresponds to the time at which α = 0.5. Application of the Hill dose-response equation to toxicity studies has provided valuable insights that facilitate the desired extension. Empirical evidence in toxicity studies suggests that, for a fixed effect level, the relationship between exposure time and the concentration of a toxic agent follows the modified Haber rule^5, 11, 23, 24^:

15 $${(C-{C}_{o})}^{\beta }t=1/\kappa $$

where *C*_*o*_ is a threshold concentration (measurable or biologically-effective) and *β* and *κ* are constants. It is convenient to peg the effect level in equation (10) at *α* = ½ by an appropriate choice of the constant *κ*. In toxicology this just means that *τ* is associated with toxicity measures such as *LC*_50_ or *LD*_50_. With α = ½ it then follows from equation (9) that *kt = θ* and substituting for the time variable in equation (10) yields

16 $$k=\kappa \theta {(C-{C}_{o})}^{\beta }\,$$

In essence, this shows that the modified Haber Rule is a consequence of the assumption that the rate constant in the Hill model differential equation has a power law concentration dependence on the active agents. Note that the parameter *κ* has dimensions of [T]^−1^[C]^−*β*^.

Substituting equation (16) back into equation (14) yields the general form of the Hill response function that covers both time and dose levels for a single active agent. In the odds form it is defined by

17 $$\alpha /(1-\alpha )={[\kappa {(C-{C}_{o})}^{\beta }t]}^{\theta }$$

This implies that the general form for the time constant in equation (14), based on equation (17), is

18 $$\,\tau ={[\kappa {(C-{C}_{o})}^{\beta }]}^{-1}$$

The equivalent dose-response form of equation (9), at a fixed test time *t*_e_, is then

19 $$\alpha /(1-\alpha )={[(C-{C}_{o})/({C}^{\ast }-{C}_{o})]}^{\beta \theta }$$

where C* is defined by the relationship

20 $${C}^{\ast }-{C}_{o}={({\rm{\kappa }}{t}_{e})}^{-1/{\rm{\beta }}}$$

Setting *C*_o_ = 0 reduces equation (19) to the conventional Hill dose-response function determined for a fixed evaluation time^44^. Finally, combining equations (19) and (20) provides

21 $$t/\tau ={[(C-{C}_{o})/({C}^{\ast }-{C}_{o})]}^{\beta }$$

### Effect of active agent mixtures

The development was limited to the case where the effect of all agents are described by Hill equations with the same identical *θ* value. In what follows, it is also assumed for convenience that there is no threshold concentration, i.e. *C*_o_ = 0.

### Independent chemical action

Note first that, if the agents do not interact from the perspective of chemical reaction kinetics, the rate expression of equation (10) implies that

22 $${k}_{mix}=\sum _{i}{k}_{i}$$

So that

23 $${\tau }_{mix}={[\sum _{i}1/{\tau }_{i}]}^{-1}$$

This means that the standard Hill response function holds for the mixture except that an effective scale parameter, defined by equation (23), is used. Note that equation (23) defines the mixture time constant as a harmonic mean over the *τ* values that would have applied had the actives been applied individually on their own. Equation (23) defines the response of the system on the assumption that the active agents act independently in a chemical rate sense. This is not the same as the conventional concept of Independent Action^10^.

### Concentration Addition

**Figure 5:**
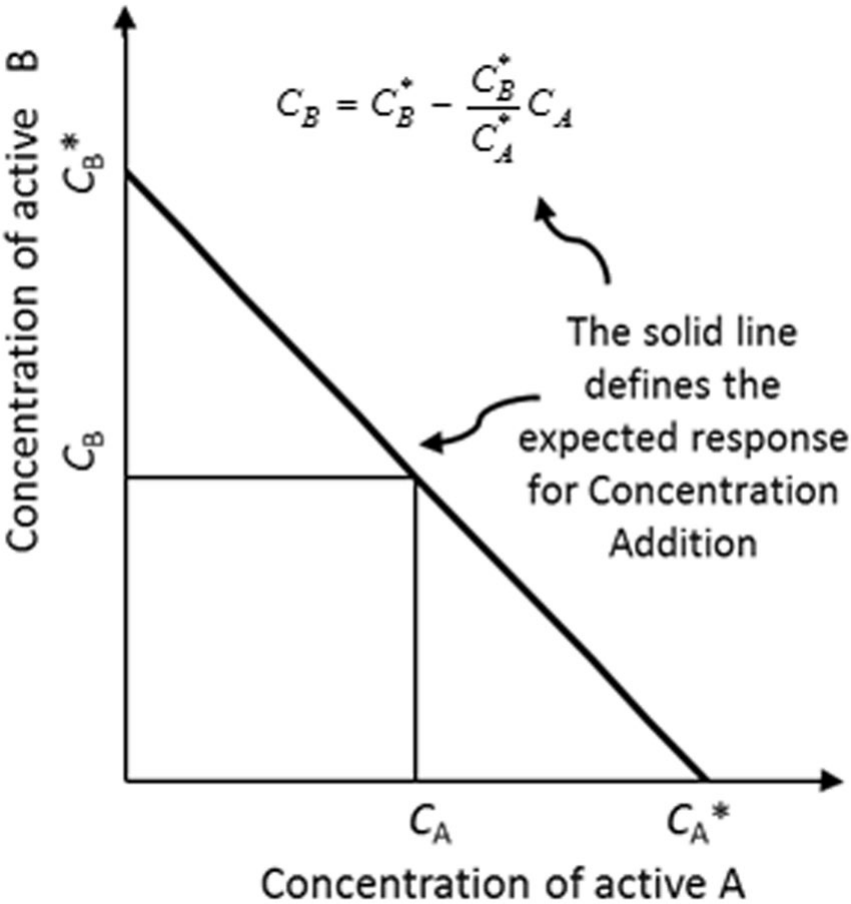
Illustrating the concept of Concentration Addition (CA) with an isobologram for a binary mixture of active agents. *C*_A_* and *C*_B_* correspond to the concentrations at which application of agents A and B lead to a specific effect, e.g. *LC*_50_ in toxicology. The straight line defines the expected effect for concentration addition.

Equation (23) is based on the assumption that the active agents act independently according to the rate expression of equation (9). This is not necessarily the case and more often than not, when dosed as a combination, they will in fact affect each other’s performance. In such scenarios it is necessary to find suitable mixing rules for either the rate constant (*k*) or the mixture time constant (*τ*). In essence a mixing rule defines a way to calculate a mixture parameter from knowledge of the parameters that apply when the individual active agents operate on their own^45^. The concept of concentration addition (CA), often applied in toxicological studies, can be used to define such a mixing rule. The CA concept is best explained by considering the isobologram presented in Fig. 5. An isobologram is a graph used for binary mixtures of active agents. The two linear dose axes define a Cartesian plane on isoboles, i.e. equi-effect curves at various concentrations or doses of the two active agents are plotted^45, 46^. The straight line isobole in Fig. 5 defines the expected response for concentration addition^45^ when two actives are administered as a mixture:

24 $${C}_{B}={C}_{B}^{\ast }-\frac{{C}_{B}^{\ast }}{{C}_{A}^{\ast }}{C}_{A}$$

This equation can be re-arranged to read:

25 $$\frac{{C}_{A}}{{C}_{A}^{\ast }}+\frac{{C}_{B}}{{C}_{B}^{\ast }}=1$$

For a mixture of agents with composition defined by the fractions *x*_A_ and *x*_B_ respectively, the required concentration to achieve the same desired effect level is then given by:

26 $$\frac{1}{{C}_{mix}^{\ast }}=\frac{{x}_{A}}{{C}_{A}^{\ast }}+\frac{{x}_{B}}{{C}_{B}^{\ast }}$$

The full dose response curve is obtained by substituting ${C}_{mix}^{\ast }$ for C* in equation (19) with *C*_o_ = 0. This also applies to equation (16) from which, after some simple algebraic manipulations, the following result is obtained for Concentration Addition (CA):

27 $${\tau }_{mix}={[{{\tau }_{A}}^{-1/\beta }+{{\tau }_{B}}^{-1/\beta }]}^{-\beta }$$

Again the standard Hill response function (equation (13)) holds except that the scale parameter for the mixture, as determined by equation (27), is used. Note that equation (27) states that *τ*_mix_ is a power mean of order −1/*β* over the individual *τ*_*i*_ values applicable when the agents were used on their own. Some special cases are of interest:

- When *β* = −1 then the rate constant is inversely proportional to the active agent concentration. This type of response is expected for a beneficial agent that improves the survival rate. In this case *τ*_mix_ is given by a summation over the individual *τ*_*i*_ values:
  28 $${\tau }_{mix}={\tau }_{A}+{\tau }_{B}$$
- When *β* = 1 it implies that the rate constant is directly proportional to the active agent concentration. This type of response is expected for a harmful agent, e.g. a toxin that decreases the survival rate. In this case *τ*_mix_ is evaluated as a harmonic mean over the individual *τ*_*i*_ values:

29 $$1/{\tau }_{mix}=1/{\tau }_{A}+1/{\tau }_{B}$$

This is equivalent to the result found in equation (23). From this it can be concluded that independent chemical action is equivalent to Concentration Addition when the power exponent of the modified modified Haber rule equals *β* = 1. The response function for the mixture of active agents can also be expressed in terms of the individual response functions:

30 $${\alpha }_{mix}/(1-{\alpha }_{mix})={\{{[{\alpha }_{A}/(1-{\alpha }_{A})]}^{1/\beta \theta }+{[{\alpha }_{B}/(1-{\alpha }_{B})]}^{1/\beta \theta }\}}^{\beta \theta }$$

### Other isoboles

Plackett and Hewlett^25, 26^ considered the deviations from the CA line and suggested the following generalization for the action of two agents administered simultaneously:

31 $${(C/{C}_{mix}^{\ast })}^{1/\lambda }={({C}_{A}/{C}_{A}^{\ast })}^{1/\lambda }+{({C}_{B}/{C}_{B}^{\ast })}^{1/\lambda }$$

This leads to the following expressions for the mixture response time constant and the mixtures odds function:

32 $${\tau }_{mix}={[{{\tau }_{A}}^{-1/\beta \lambda }+{{\tau }_{B}}^{-1/\beta \lambda }]}^{-\beta \lambda }$$

33 $${\alpha }_{mix}/(1-{\alpha }_{mix})={\{{[{\alpha }_{A}/(1-{\alpha }_{A})]}^{1/\lambda \beta \theta }+{[{\alpha }_{B}/(1-{\alpha }_{B})]}^{1/\lambda \beta \theta }\}}^{\lambda \beta \theta }$$

where *α*_*A*_ and *α*_*B*_ are the expected responses for the agents acting on their own.

## Experimental Methods

### Materials

Pure triple-distilled sunflower oil was obtained from Sunfoil. The antioxidants considered were tetrakis[methylene(3,5-di-t-butyl-4-hydroxyhydrocinnamate)]methane (Anox 20 ex Addivant) a hindered phenolic antioxidant, poly(1,2-dihydro-2,2,4-trimethylquinoline) (Orox PK ex Orchem) an amine-type antioxidant and tris(nonylphenyl) phosphite (Naugard P ex Chemtura) a phosphite-type antioxidant. The first two antioxidants are classed as primary antioxidants while the third is classed as a secondary antioxidant. The antioxidants were added to the biodiesel at different loadings up to a maximum of 0.25 wt.%.

### Biodiesel preparation

The biodiesel was prepared using alkali-catalysed methanolysis. Potassium hydroxide was used as the catalyst and small batches of 500 mL were made. The full procedure is described elsewhere^47^.

### Biodiesel characterization

The FAME analysis was performed at Analytical Services, Food and Beverage Laboratory, CSIR, Pretoria using an Agilent 6890 GC-FID. An Agilent J&W GC column CP-SIL 88 was used for the separation of the FAME’s. Quantification was performed by internal standard calibration using methyl heptadecanoate. The FAME content was computed according to EN 14103 and Ruppel and Huybrighs^48^ with the sum of all the peaks from the methyl myristate (C_14_) peak up to that of the methyl ester of C_24:1_ accounted for. Identification of the FAMEs in the biodiesel samples was accomplished by comparing their retention times to a Supelco FAME reference mixture containing 37 components. Additional biodiesel physical properties were determined, using standard procedures, by Bio Services CC, Randburg, South Africa. These properties included free glycerine, methanol content, water content, acid value, iodine value, and flash point. The properties of the biodiesel used in this study are summarised in Table 2. It met the EN 14214 specifications including the requirement that the ester content should not be lower than 96.5%.

**Table 2:** List of biodiesel (FAME) properties made from sunflower oil.

Property	Value	Units
FAME (ester content)	98.3	wt.%
FAME composition: (%)		wt.%
Methyl palmitate, C16:0	6.73	
Methyl stearate, C18:0	6.63	
Methyl oleate, C18:1	22.4	
Methyl linoleate, C18:2	62.0	
Methyl linolenate, C18:3	0.22	
Other methyl esters	2.02	
Density at 15 °C	888	kg m^−3^
Viscosity at 40 °C	4.6	mm^2^ s^−1^
Flash point	170	°C
Water content	0.04	%
Acid value	0.1	mg KOH g^−1^
Methanol content	0	wt.%
Iodine value	118	—
Free Glycerol	0.01	wt.%
Appearance	clear	—

### Oxidative stability

The effect of antioxidant concentration on the progression of oxidative degradation was determined by spiking the biodiesel with different amounts of neat antioxidant, or mixtures of antioxidants as summarised in Table 1. Triplicate measurements were done on a total of 22 different compositions including the neat biodiesel. The oxidation stability of the neat biodiesel as well as the stabilised biodiesel samples was determined using a Metrohm 895 Professional PVC Thermomat. All the biodiesel oxidation stability analyses were done using the Rancimat method described in European Standard EN 14112. The oxidation test was done at a constant temperature of 110 °C and the airflow rate was set at 10 L h^−1^. Biodiesel samples (3.00 g) were weighed into the reaction vessels, and placed in the heated cellblock. Air was passed through the sample and then through a measuring vessel containing 50 mL of deionised water. The volatile acids formed during the oxidation process are absorbed in this water and increase its conductivity. This increase in conductivity is measured as a function of time in the Rancimat method.

### Data reduction

In this study, it was assumed that the conductivity vs. time curves (*σ* = *σ*(*t*)) could be represented by equation (34):

34 $$\sigma (t)={\sigma }_{\min }+mt+P\alpha (t)$$

where *σ*(*t*) is the experimental conductivity vs. time curve; *σ*_*min*_ is the conductivity offset at time *t* = 0; *m* is the slope of the initial portion of the conductivity curve; *P* is a proportionality constant and α(*t*) is the response function. Note that the parameter *m* in equation (34) compensates for any linear signal drift over the full measurement time. The response function *α*(*t*) should be able to adequately represent the experimental data over the full measurement range. Model parameters were obtained by least square regression using Solver**-**based Microsoft Excel programming^49^. This showed that the Hill expression, equation (13), was adequate for the present data set with *θ* = 3.45 ± 0.10 assumed to be the same for all data sets.
